# Reconfiguration of Functional Dynamics in Cortico-Thalamo-Cerebellar Circuit in Schizophrenia Following High-Frequency Repeated Transcranial Magnetic Stimulation

**DOI:** 10.3389/fnhum.2022.928315

**Published:** 2022-07-25

**Authors:** Huan Huang, Bei Zhang, Li Mi, Meiqing Liu, Xin Chang, Yuling Luo, Cheng Li, Hui He, Jingyu Zhou, Ruikun Yang, Hechun Li, Sisi Jiang, Dezhong Yao, Qifu Li, Mingjun Duan, Cheng Luo

**Affiliations:** ^1^The Clinical Hospital of Chengdu Brain Science Institute, MOE Key Lab for Neuroinformation, School of Life Sciences and Technology, University of Electronic Science and Technology of China, Chengdu, China; ^2^Department of Psychiatry, The Clinical Hospital of Chengdu Brain Science Institute, University of Electronic Science and Technology of China, Chengdu, China; ^3^Department of Neurology, First Affiliated Hospital of Hainan Medical University, Haikou, China; ^4^University of Science and Technology Beijing, Beijing, China; ^5^Research Unit of Neuroinformation, Chinese Academy of Medical Sciences, Chengdu, China

**Keywords:** schizophrenia, functional connectivity, temporal variability, transcranial magnetic stimulation, fMRI

## Abstract

Schizophrenia is a serious mental illness characterized by a disconnection between brain regions. Transcranial magnetic stimulation is a non-invasive brain intervention technique that can be used as a new and safe treatment option for patients with schizophrenia with drug-refractory symptoms, such as negative symptoms and cognitive impairment. However, the therapeutic effects of transcranial magnetic stimulation remain unclear and would be investigated using non-invasive tools, such as functional connectivity (FC). A longitudinal design was adopted to investigate the alteration in FC dynamics using a dynamic functional connectivity (dFC) approach in patients with schizophrenia following high-frequency repeated transcranial magnetic stimulation (rTMS) with the target at the left dorsolateral prefrontal cortex (DLPFC). Two groups of schizophrenia inpatients were recruited. One group received a 4-week high-frequency rTMS together with antipsychotic drugs (TSZ, *n* = 27), while the other group only received antipsychotic drugs (DSZ, *n* = 26). Resting-state functional magnetic resonance imaging (fMRI) and psychiatric symptoms were obtained from the patients with schizophrenia twice at baseline (*t1*) and after 4-week treatment (*t2*). The dynamics was evaluated using voxel- and region-wise FC temporal variability resulting from fMRI data. The pattern classification technique was used to verify the clinical application value of FC temporal variability. For the voxel-wise FC temporary variability, the repeated measures ANCOVA analysis showed significant treatment × time interaction effects on the FC temporary variability between the left DLPFC and several regions, including the thalamus, cerebellum, precuneus, and precentral gyrus, which are mainly located within the cortico-thalamo-cerebellar circuit (CTCC). For the ROI-wise FC temporary variability, our results found a significant interaction effect on the FC among CTCC. rTMS intervention led to a reduced FC temporary variability. In addition, higher alteration in FC temporal variability between left DLPFC and right posterior parietal thalamus predicted a higher remission ratio of negative symptom scores, indicating that the decrease of FC temporal variability between the brain regions was associated with the remission of schizophrenia severity. The support vector regression (SVR) results suggested that the baseline pattern of FC temporary variability between the regions in CTCC could predict the efficacy of high-frequency rTMS intervention on negative symptoms in schizophrenia. These findings confirm the potential relationship between the reduction in whole-brain functional dynamics induced by high-frequency rTMS and the improvement in psychiatric scores, suggesting that high-frequency rTMS affects psychiatric symptoms by coordinating the heterogeneity of activity between the brain regions. Future studies would examine the clinical utility of using functional dynamics patterns between specific brain regions as a biomarker to predict the treatment response of high-frequency rTMS.

## Introduction

Schizophrenia is considered as a serious mental disease resulting in huge and severe effects on individuals and society. It is characterized by a heterogeneous range of positive symptoms, such as hallucinations, delusions, and thought disturbances, and negative symptoms, such as social/affective deficits and cognitive impairment, including working memory and executive functioning deficits (Andreasen, 1982). Treatment schemes based on antipsychotics have been continuously developed and achieved good results for positive symptoms. However, for negative symptoms, the effects of antipsychotic treatment were relatively weak. Various functional and social deficits in schizophrenia were strongly associated with negative symptoms (Ventura et al., 2015). Previous placebo-controlled experimental studies have mostly focused on positive symptoms in patients with schizophrenia, whereas treatment of negative symptoms can truly help patients return to society (Buchanan, 2007). Therefore, investigating the underlying mechanisms for alleviating negative symptoms might contribute to explore more effective treatment protocol.

Non-invasive brain stimulation technology mainly includes transcranial magnetic stimulation (TMS) (Rabany et al., 2014; Lefaucheur et al., 2020; Ji et al., 2021), transcranial direct current stimulation (tDCS) (Lefaucheur et al., 2017; Valiengo et al., 2020), deep brain stimulation (DBS) (Lippmann et al., 2021), transcranial alternating current stimulation (tACS) (Ahn et al., 2019), and transcranial ultrasound stimulation (TUS) (Zhang et al., 2021). The underlying neurobiological cause for the development of schizophrenia, referred to as “dysconnectivity” syndrome, is the failure of coordination between multiple brain regions (Stephan et al., 2009), and these non-invasive brain stimulation methods shed new light on coordinating abnormal connections between brain regions. The TMS pulses induced electric currents under the localized areas of the stimulation site through a rapidly changing magnetic field, leading to changes in local neuronal activation and functional connectivity (FC) between the distributed brain regions (Allen et al., 2007; Chen et al., 2013). Therefore, the repeated TMS (rTMS) was considered as a novel and safe therapeutic option for the treatment of some refractory symptoms in schizophrenia that were not effectively controlled with antipsychotics, such as negative symptoms and cognitive impairment (Aleman et al., 2018). Evidence from previous studies has demonstrated that rTMS could effectively relieve the drug-refractory symptoms of schizophrenia, but the results were inconsistent (Wobrock et al., 2015; Li et al., 2016; Gan et al., 2021). For example, one study found that high-frequency rTMS alleviated the negative symptoms of schizophrenia and the effect lasted for at least 4 weeks (Gan et al., 2021). Similarly, some researchers found that the negative symptoms in patients with schizophrenia were improved after 8 weeks of treatment with 10 Hz high-frequency rTMS, suggesting that high-frequency rTMS showed a delayed effect on the negative symptoms (Li et al., 2016). However, Wobrock et al. (2015) found no difference in the improvement of negative symptoms in schizophrenia between 10 Hz rTMS and sham rTMS treatments to the left dorsolateral prefrontal cortex (DLPFC). Several factors could explain the differences in these studies, including heterogeneity in schizophrenia, diversity of disease states, and differences in rTMS operations. Importantly, several meta-analyses have demonstrated the therapeutic effect of rTMS on negative symptoms in schizophrenia, and suggest that the optimal rTMS parameter for the treatment of negative symptoms is 20 times rTMS treatment over 4 weeks, targeting left DLPFC (Shi et al., 2014; Jiang et al., 2019).

Taken together, it was essential to investigate the underlying mechanisms of high-frequency rTMS in the functional activity of the brain in schizophrenia. Considering to enhance the “hypofrontality” of the dominant prefrontal cortex for improving negative symptoms and cognitive dysfunction in schizophrenia, most studies have attempted to deliver high-frequency rTMS to the left or bilateral prefrontal cortex (Barch et al., 2001; Mehta et al., 2019). Furthermore, high-frequency rTMS can induce a continuous increase in regional cerebral blood flow in bilateral frontal, limbic, and paralimbic regions in depressed patients (Speer et al., 2000). In contrast to the study of brain activity, the recent trends are studying the responses of FC to TMS, and most studies focused on static FC (Beynel et al., 2020). The commonly adopted FC approach assumed that brain networks are statically configured. This assumption might impact FC sensitivity to rTMS perturbation effects, leading to inconsistent results and discouraging the application of FC in exploring the mechanisms of rTMS. Compared with FC, dynamic FC (dFC) provided a potential tool to capture sensitive changes that occur in psychiatric or neurologic disorders between brain regions (Dong et al., 2019). However, only a few studies have focused on dFC responses to TMS (Tang et al., 2019; Vergara et al., 2021). For example, Vergara et al. (2021) found that TMS induced functional connectivity oscillations in the brain using a dynamic functional network connection. Another study combined TMS and fMRI to track the spatiotemporal dynamics of TMS after effects within the fronto-limbic network (Tang et al., 2019). The dFC method might be useful to assess the reconfiguration of FC temporal variability following high-frequency rTMS in schizophrenia, and provide evidence for the effectiveness of high-frequency rTMS in the treatment of schizophrenia.

As a non-drug therapy strategy, rTMS has a broad application prospect in the treatment or cure of schizophrenia. In our study, we adopted a time-varying approach to comprehensively characterize the regulatory effect of high-frequency rTMS on FC temporal variability in schizophrenia. Beyond the previous stationary characterization, it might provide some quantitative insights into the mechanisms of rTMS to enhance our understanding of the relationship between high-frequency rTMS treatment and psychiatric symptoms.

## Materials and Methods

### Subjects

Fifty-nine inpatients with schizophrenia were recruited from the Clinical Hospital of Chengdu Brain Science Institute (CBSI). All patients met the ICD-10 diagnostic criteria for schizophrenia. Clinical symptomatic severity was evaluated by the Positive and Negative Syndrome Scale (PANSS). Patients were included if their T1 images at baseline showed no organic lesions, negative symptom sub-score equal to or greater than 21, and positive symptom sub-score equal to or smaller than 24 on the PANSS with no distinct auditory hallucinations. The exclusion criteria included a history of other mental disorders and drug abuse, recent suicidal tendencies, TMS and MRI contraindications, and neurological disorders in patients or their first-degree relatives. A set of matched MRI data of 24 healthy controls was recruited as the healthy control group (HC group), which was used as a healthy reference, and the data were also obtained from the Clinical Hospital of CBSI research database. The HCs were screened for a history of medical or neuropsychiatric illness, as well as for major neurological or psychiatric illness in their first-degree relatives.

### Design

To examine the effect of high-frequency rTMS treatment on patients with schizophrenia, our study conducted a longitudinal experimental design. The design consisted of two time-point tests, including an fMRI scan and PANSS scale test at baseline (*t1*) and the same tests about 1 month later (*t2*). Subsequently, we divided the patients into two groups according to the treatment strategy. Thirty patients (men: 23; women: 7) received high-frequency rTMS treatment for 4 weeks in combination with a stable dosage of antipsychotics (TSZ group); the remaining 29 patients received antipsychotic medication only (DSZ group). All patients had to remain on the same medication treatment throughout the duration of the study. All participants and/or their guardians should be aware of the purpose as well as the procedure of our study and were required to provide written consents to be included in this study. The Ethics Committee of the Clinical Hospital of Chengdu Brain Science Institute approved the study protocol.

### Repeated Transcranial Magnetic Stimulation Protocol

In this study, rTMS treatment was delivered using a YRDCCY-1 stimulator (Yiruide Medical Equipment New Technology Co., Ltd., Wuhan, China) with a figure-8-shaped coil (B9076). The loop coil provides stimulation tangentially to the plane of the skull, and the middle position of the loop coil is aligned with the stimulation target. For the stimulation target (left DLPFC), the tip of the intersection of the two coil loops was lined up with the F3 sites of the 10–20 electroencephalogram system (Turriziani et al., 2010). The rTMS was applied, using trains of 10 Hz frequency and an intensity of 110% of the rest motor threshold (RMT). The RMT was individually determined before the stimulus according to a five-step procedure (Schutter and van Honk, 2006). Participants received 20 rTMS treatment sessions in 4 weeks, targeting left DLPFC (Shi et al., 2014; Jiang et al., 2019). Each session consisted of 1,000 pluses (20 trains with 50 pulses duration, 5 s per train, and an interval of 30 s). The entire session lasted 11 min and 40 s. The treatment procedure was only applied to the TSZ group.

### MRI Data Acquisition and Preprocessing

For all three groups, we acquired the fMRI data of all the participants at baseline. For the two groups of patients, there was another fMRI acquisition after 1 month. The experiments were performed on a 3-Tesla MRI scanner (GE DISCOVERY MR750, United States) at the University of Electronic Science and Technology of China (UESTC). During scanning, we used a foam head pad and ear plugs to reduce the head motion and scanning noise, respectively. The resting-state functional MRI data were acquired using gradient-echo echo-planar imaging (EPI) sequences. The parameters included repetition time (TR) = 2,000 ms, echo time (TE) = 30 ms, flip angle (FA) = 90°, image matrix = 64 × 64, field of view (FOV) = 240 × 240 mm^2^, and slice thickness/gap = 4 mm/0.4 mm), with an eight-channel phased-array head coil. All subjects underwent a 510 seconds resting-state scan to yield 255 volumes (35 slices per volume) and were required to be relaxed with their eyes open and staring at a cross on the screen.

The preprocessing of resting-state functional data was performed using the NIT toolbox (Dong et al., 2018). The first five volumes were discarded for the magnetization equilibrium. The preprocessing steps were as follows: (1) slice timing correction; (2) head motion correction; (3) normalization: the functional data were spatially normalized (3 × 3 × 3 mm^3^) to the MNI template; (4) smoothed by Gaussian kernel (FWHM = 8 mm); (5) temporal filtering was performed at band-pass 0.01–0.08 Hz; and (6) nuisance signals were regressed out, including white matter (WM), cerebrospinal fluid (CSF), linear trend, and 12-motion parameters ( x-, y-, z-translations, three rotations, and their derivatives), except for the global signal due to a recent excellent study that demonstrated that altered global brain signal was observed in patients with schizophrenia, which may underlie profound alterations in the neural information flow in patients with schizophrenia, regressing out global mean signal can distort between-group comparisons of inter-regional correlation (Yang et al., 2014). In addition, a recent study also demonstrated that head motion has a substantial impact on FC (Power et al., 2012). Thus, any subjects who had a maximum translation in any of the cardinal directions larger than 3.0 mm or a maximum rotation larger than 3.0° were excluded from the subsequent analysis. In addition, frame-wise displacement (FD) was evaluated in the three groups as suggested by Power et al. (2012).

### Functional Connectivity Temporal Variability Mapping

The preprocessed blood oxygen level dependence (BOLD) time courses of the voxel of stimulating target (left DLPFC with MNI coordinate: −51, 21, and 18) and its nearest six neighbors were extracted and averaged for each subject. We performed the same analysis on the data obtained for both groups of patients at *t1* and *t2*. For the voxel-wise FC temporal variability, the left DLPFC was selected as the seed point to calculate FC temporal variability with all the other voxels in the brain. In detail, we used the sliding windows approach to investigate the FC temporal variability. For the optimal window length, recent research has shown that the minimum window length should be no less than 1/fmin (Leonardi and Van De Ville, 2015). Thus, the time courses were segmented into 50-TR windows (fmin = 0.01 Hz) with a sliding step length of 1 TR, i.e., a total of 201 windows were divided from 250 time points of resting-state data. Then Pearson correlation was calculated in each window between the left DLPFC and all the other brain voxels. We defined the FC temporal variability of left DLPFC as follows:


$$I=\frac{1}{m-1}\,\sum_{k=2}^{m}{\left(r_{k}-r_{k-1}\right)}^{2}$$

where *m* represents the number of the sliding windows (m = 201 in this study) and *r_k* represents the Pearson correlation between left DLPFC and each other voxel over time window *k* (*k* = 2, 3, …, *m*).

In addition, the ROI-wise FC temporal variability induced by high-frequency rTMS was further explored. Pursuant to the statistical analysis results of the first-level analysis, the clusters with significant differences were selected as ROI. Considering the importance of the thalamus in the pathological study of schizophrenia, we adopted a more precise atlas of thalamus subregions (Fan et al., 2016). It should be noted that left DLPFC is also retained as an ROI. The time course of each ROI was defined as the mean of the time course of the peak value voxel and its nearest six neighbors. Then, in each window, we calculated the Pearson’s correlation coefficient between the BOLD time courses of each pair of ROIs. The FC temporal variability *I* between each pair of ROIs was determined according to the above-mentioned formula. Finally, for each subject, a *m* × *m* (*m* = number of ROIs) FC temporal variability matrix was generated.

### Statistical Analysis

The demographic and psychometric data were analyzed using SPSS 28.0. For DSZ and TSZ groups, the two-sample *t*-tests were used to compare age and years of education, and the chi-square test was performed to compare the differences in gender. To assess the therapeutic effect, repeated measures ANCOVA was performed to compare the PANSS scores between the TSZ and DSZ groups. The time course and treatment differences in relation to the changes in clinical symptoms were evaluated by means of a mixed-effects model for repeated measures analysis with the main effects of treatment and time and a treatment × time interaction adjusted for age, gender, illness duration, education level, and antipsychotic dosage. Additionally, the remission ratio of the PANSS negative scores related to treatment was calculated as follows:


$${\text{RR}}_{\text{negative}}=\frac{{\text{PANSS}}_{\mathrm{negative}\,\_\,\mathrm{t1}}-{\text{PANSS}}_{\mathrm{negative}\,\_\,\mathrm{t2}}}{{\text{PANSS}}_{\mathrm{negative}\,\_\,\mathrm{t1}}}$$

where PANSS_*negative*_*t1*_ and PANSS_*negative*_*t2*_ refer to the negative PANSS scores at *t1* and *t2*, respectively, and RR represents the remission ratio. A similar formula was used to calculate PANSS positive, general, and total scores. Then, a two-sample *t*-test was performed to compare the remission ratios of TSZ and DSZ PANSS scores.

For the voxel-wise FC temporal variability, repeated measures ANCOVA was performed to assess the between-subject factor group (rTMS + Drug vs. Drug) and the within-subject factor time (*t1* vs. *t2*) while controlling for age, gender, illness duration, education level, and antipsychotic dosage. To examine whether rTMS+Drug and Drug have different modulating effects on FC temporal variability, we focused on the interaction effect between the group and time. *Post-hoc* analysis was performed using a two-sample *t*-test to evaluate the group differences at *t1* and *t2*, and two paired *t*-tests were used to compare the differences between *t1* and *t2* for each group. In addition, each patient group was compared with the HC group by a two-sample *t*-test. All analyses were performed for multiple comparisons correction using a height threshold (*p* = 0.005) and an extent threshold based on Gaussian Random Field Theory (*P*-corrected = 0.05) (Chen et al., 2017; Huang et al., 2018). Regions that demonstrated a significant interaction effect were selected for ROI-wise FC temporal variability analysis. The statistical approach of ROI-wise FC temporal variability was consistent with the voxel-wise analysis.

### Relationships Between Functional Connectivity Temporal Variability Alterations and Clinical Features

The ROI-wise FC temporal variability patterns were extracted from the ROIs with significant interaction effects. The alteration in FC temporal variability induced by high-frequency rTMS was defined as the amplitude difference between the *t2* and *t1* time points divided by the *t1* amplitude. As the results did not conform with normal distribution, we used Spearman correlation analysis to investigate the relationships between alterations in FC temporal variability and remission ratio of the PANSS scores, after controlling for age, sex, illness duration, education level, and drug dosage.

### Pattern Classification Analyses

Pattern classification was conducted using the support vector regression (SVR) implementation from the scikit-learn library (v0.24.1^1^). In the present study, SVR was applied to explore the competence of the ROI-wise FC temporal variability of patients at *t1* to predict the high-frequency rTMS treatment response as reflected by the remission ratios of the PANSS total and subscale scores. In order to optimize the parameters of the SVR model, the leave-one-out cross-validation method was used to search for the optimal parameters. A Grid search was performed on predefined parameter spaces. To evaluate the prediction model, the Pearson correlation coefficient [*r*_*(predicted, actual)*_] was calculated between actual remission ratios and estimated remission ratios of the PANSS total and subscale scores.

### Relationships Between Functional Connectivity Temporal Variability and Functional Connectivity

Most of the previous studies have analyzed the effect of high-frequency rTMS on the FC in patients with schizophrenia. To further illustrate the significance of FC temporal variability in our study, we explored whether the FC temporal variability change was accompanied by increased or decreased FC. Interestingly, our results showed that there was a significant negative correlation between FC temporal variability and FC between brain regions at the group level in both the schizophrenia group and the HC group (Supplementary Figure 1), that is, the decrease in FC temporal variability was accompanied by the increase in FC. The detailed information and description are given in Supplementary Material.

## Results

### Demographic and Clinical Data

Due to excessive head motion or not being able to complete the experiment, six patients (three in TSZ and three in DSZ) were excluded from our study. Fifty-three patients with schizophrenia and 24 HCs were included in the subsequent analysis. In this study, there was no significant difference between the two groups in terms of gender, age, years of education, duration of illness, and dosage of antipsychotic drugs. The demographic information is displayed in Table 1.

**TABLE 1:** Participants’ fundamental information.

Characteristic	TSZ (*n* = 27) Mean (SD)	DSZ (*n* = 26) Mean (SD)	*p* value	HC (*n* = 24) Mean (SD)
Gender (female/male)^a^	7/20	6/20	0.811	12/12
Age (years)^b^	46.7 (11.73)	45.85 (8.41)	0.755	44.96 (4.46)
Education (years)^b^	10.04 (2.95)	11.69 (3.23)	0.062	9.68 (2.75)
Illness duration (months)^b^	17.07 (8.79)	19.56 (8.46)	0.302	–
Chlorpromazine equivalents (mg/d)^b^	291.63 (104.92)	317.96 (117.00)	0.119	–
Baseline PANSS score^b^				
Positive	11.70 (4.16)	11.62 (4.88)	0.945	–
Negative	23.56 (6.70)	21.77 (7.92)	0.388	–
General	30.41 (6.13)	28.92 (6.21)	0.394	–
Total	65.67 (12.70)	62.31 (16.40)	0.416	–
**1-month follow up PANSS score^b^**				
Positive	9.96 (3.44)	10.23 (4.03)	0.799	
Negative	18.07 (5.06)	20.19 (7.53)	0.242	
General	25.81 (6.09)	27.00 (5.32)	0.463	
Total	53.85 (11.69)	57.42 (14.18)	0.330	

*PANSS, Positive and Negative Syndrome Scale; TSZ, the group receiving antipsychotic drugs and rTMS; DSZ, the group only received antipsychotic drugs; HC, healthy control; SD, standard deviation.*

*^a^Chi-square tests.*

*^b^Two-sample t-test.*

Through a repeated measures ANOVA analysis, we observed significant treatment x time interaction in negative, general, and total scores of the PANSS scores (Table 2). All symptoms showed significant main effects of time, but none showed significant main effects of treatment. *Post-hoc* analysis revealed a significant decrease in the PANSS scores in both the TSZ group and the DSZ group. No significant difference between the TSZ and DSZ groups at either *t1* or *t2* was found for any of the features of the psychiatric symptoms. Importantly, the remission ratios of negative (*t* = 4.031, *p* < 0.001), general (*t* = 2.958, *p* = 0.005), and total scores (*t* = 3.826, *p* < 0.001) of PANSS in the TSZ group were significantly higher than those observed in the DSZ group (Table 2).

**TABLE 2:** Repeated measured ANCOVA on Positive and Negative Syndrome Scale (PANSS) scores and the test of remission ratio between TSZ and DSZ groups.

PANSS scores	Interaction effects	Time main effects	*Post-hoc* (paired *t*-test)		PANSS remission ratio*
			TSZ	DSZ	
Positive	0.683	0.001	0.024	0.006	0.919
Negative	0.001	<0.001	<0.001	0.027	<0.001
General	0.012	<0.001	<0.001	0.015	0.005
Total	0.004	<0.001	<0.001	0.005	<0.001

*TSZ, the group received rTMS together with antipsychotic drugs; DSZ, the group only received antipsychotic drugs.*

*The two-sample t-test of PANSS remission ratio between TSZ and DSZ groups.

### Significant Functional Connectivity Temporal Variability Change for Voxel-Wise Analysis

**FIGURE 1:**
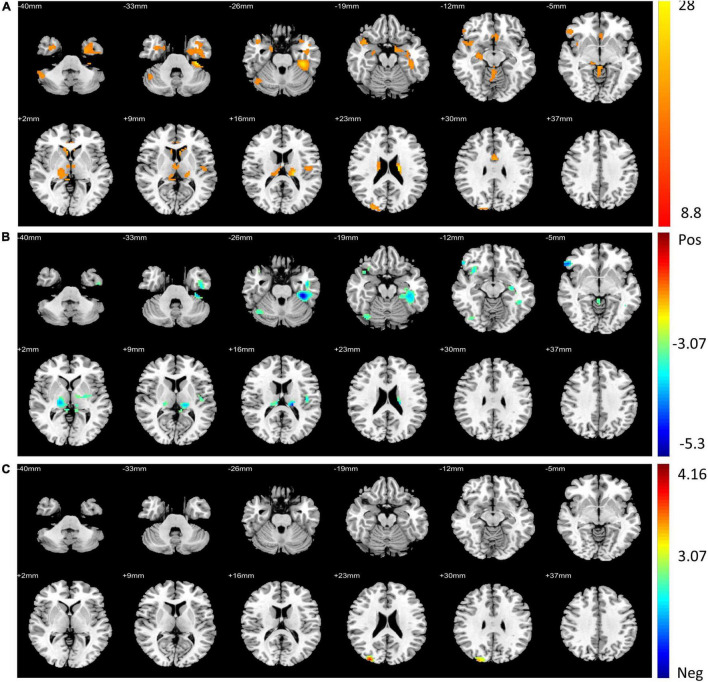
Regions with significant differences in voxel-wise functional connectivity (FC) temporal variability between post- and pre-repeated transcranial magnetic stimulation (pre-rTMS) interventions. **(A)** Regions with significant treatment and time interaction effects in repeated measures ANCOVA. **(B)** The paired *t*-test of the TSZ group showed significant differences in post-repeated transcranial magnetic stimulation (post-rTMS) compared with pre-rTMS. **(C)** The paired *t*-test of the DSZ group showed significant differences between post- and pre-rTMS interventions. The threshold was set as *p* < 0.005 and volume > 600 mm^3^.

For the voxel-wise FC temporal variability, the repeated measures ANCOVA analysis showed that the brain network with significant treatment × time interaction effects on the FC temporal variability of the left DLPFC mainly included default mode network (DMN) (bilateral precuneus and left anterior cingulate cortex), sensorimotor network (SMN) (left rolandic operculum, right postcentral, and paracentral lobule), fronto-parietal network (FPN) (orbital inferior frontal gyrus), visual network (VN) (left fusiform and right superior occipital gyrus), limbic network (LN) (left inferior temporal gyrus, right superior temporal pole, and middle temporal pole), basal ganglia network (BGN) (bilateral thalamus, parahippocampal gyrus, and right hippocampus), and cerebellum network (Figure 1A and Table 3). These brain regions are mainly located in the cortical-thalamic-cerebellar circuit (CTCC). The *post-hoc* paired *t*-test analysis revealed significantly decreased FC temporal variability in a wide range of brain regions in the TSZ group compared to *t1*, including DMN, SMN, FPN, LN, BGN, and cerebellum, but no increased regions were found (Figure 1B). In contrast, the results of the paired *t*-test in the DSZ group only showed increased FC temporal variability between left DLPFC and left precuneus, and no decreased regions were found (Figure 1C). Overall, intervention with high-frequency rTMS results in reduced FC temporal variability between left DLPFC and other brain regions in schizophrenia. Meanwhile, compared with the HC group, both DSZ and TSZ groups showed increased FC temporal variability at *t1*, and the abnormal increase in FC temporal variability in the TSZ group was alleviated at *t2*, but not in the DSZ group (Supplementary Figures 2, 3). According to the above-mentioned results, regions with significant interaction effects were chosen as ROIs to perform ROI-wise FC temporal variability analyses (Table 3).

**TABLE 3:** Significant interaction effects in voxel-wise functional connectivity (FC) temporal variability analysis.

	MNI coordinates				
Brain region	x	y	z	F-value	Cluster size (voxels’ number)
**Cerebral regions**					
Temporal_Inf_L	−45	−10	−35	10.2	146
Fusiform_L	−36	−30	−26	24.6	130
Precuneus_L	−6	−70	57	11.1	62
Cingulum_Ant_L	−3	30	−1	13.1	30
ParaHippocampal_L	−14	−3	−17	12.1	30
Rolandic_Oper_L	−44	−14	14	13.0	27
Frontal_Inf_Orb_R	50	33	−7	12.0	30
Precuneus_R	3	−71	56	15.6	75
ParaHippocampal_R	18	−4	−26	12.0	32
Hippocampal_R	25	−12	−14	10.8	36
Postcentral_R	12	−45	81	15.5	63
Paracentral_Lobule_R	6	−35	79	14.8	40
Occipital_Sup_R	27	−88	24	13.1	41
Temporal_Pole_Sup_R	41	12	−22	14.8	54
Temporal_Pole_Mid_R	33	4	−35	12.8	27
**Thalamus**					
rostral temporal thalamus_L	−3	−9	6	10.6	42
caudal temporal thalamus_L	−15	−24	15	15.6	−
rostral temporal thalamus_R	4	−8	5	12.7	100
caudal temporal thalamus_R	9	−29	9	11.3	−
posterior parietal thalamus_R	16	−23	1	13.2	−
lateral prefrontal thalamus_R	12	−18	0	9.9	−
**Cerebellar regions**					
Vermis_3	1	−37	−9	12.7	26
Vermis_4_5	−2	−46	−14	9.0	33
Cerebelum_6_L	−36	−32	−29	23.3	40
Cerebelum_9_L	−6	−45	−36	10.1	26
Cerebelum_Crus1_R	46	−52	−35	10.6	46
Cerebelum_Crus2_R	53	−50	−42	12.2	53

### Significant Functional Connectivity Temporal Variability Change for ROI-Wise Analysis

**FIGURE 2:**
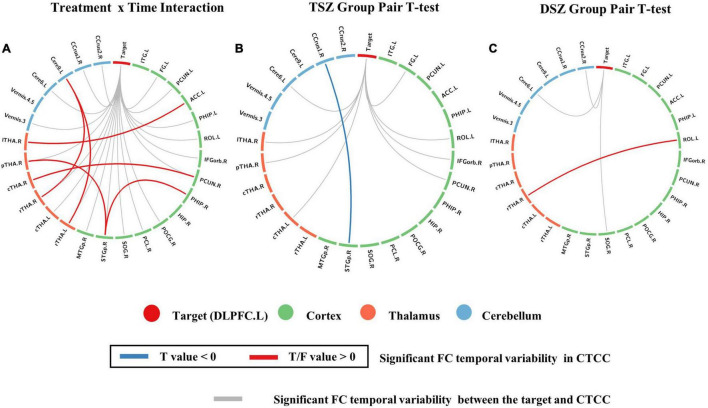
Regions with significant differences in ROI-wise functional connectivity (FC) temporal variability between post- and pre-rTMS interventions. **(A)** ROIs with significant treatment and time interaction effects in repeated measures ANCOVA. **(B)** The paired *t*-test of the TSZ group showed a significant difference between post- and pre-rTMS interventions. **(C)** The paired *t*-test of the DSZ group showed a significant difference between post- and pre-rTMS interventions. The threshold was set as *p* < 0.005.

**FIGURE 3:**
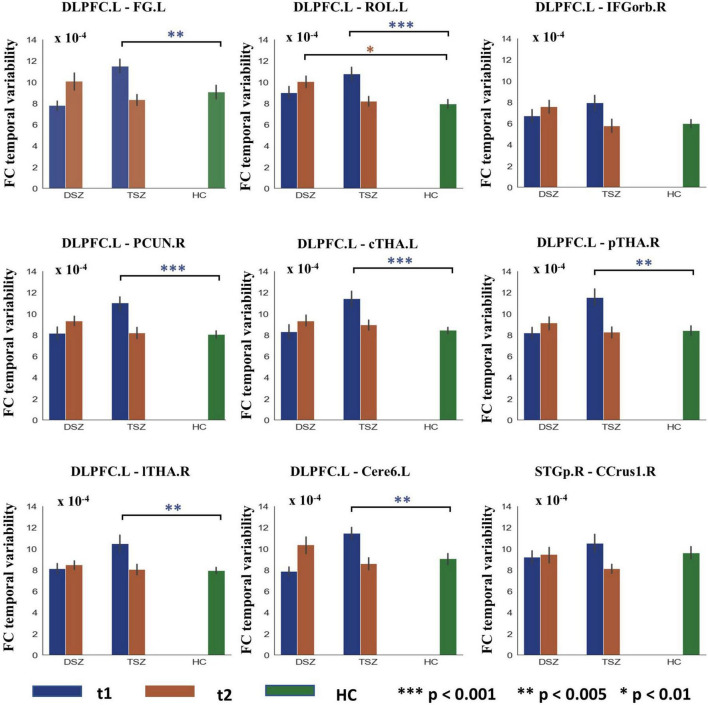
Group differences in ROI-wise functional connectivity (FC) temporal variability between TSZ, DSZ, and HC groups at post- and pre-rTMS interventions. Different numbers of * indicate different levels of significance. *p < 0.01; **p < 0.005; ***p < 0.001. DLPFC.L, left dorsolateral prefrontal cortex. Other abbreviations for ROIs can be found in Supplementary Table 1.

For the ROI-wise FC temporal variability, the repeated measures ANCOVA analysis showed that there was a significant treatment x time interaction effect on the FC temporal variability within cortex and CTCC (*p* < 0.005, Figure 2A and Table 4). The *post-hoc* analyses revealed that only decreased FC temporal variability was found in TSZ group between left DLPFC and cerebellum, thalamus, and cortical regions (Figure 2B and Table 4). Furthermore, in the DSZ group, increased FC temporal variability was found, including between left DLPFC and right superior occipital gyrus, cerebellum, thalamus, and left rolandic operculum (Figure 2C and Table 4). Two-sample *t*-test showed that compared with the HC group, the TSZ group showed an abnormal increase in FC temporal variability between brain regions at *t1*, which tended to normalize after high-frequency rTMS intervention. However, there was a trend of abnormal increase in the DSZ group (Figure 3).

**TABLE 4:** The *post-hoc* paired *t*-test analysis for TSZ and DSZ groups in ROI-wise FC temporal variability analysis.

ROI	ROI	Paired *t*-test	
		*T*-value	*P*-value
**TSZ group (*t2* vs. *t1*)**			
DLPFC_L	Fusiform_L	4.53	<0.001
DLPFC_L	Rolandic_Oper_L	3.74	<0.001
DLPFC_L	Frontal_Inf_Orb_R	3.97	<0.001
DLPFC_L	Precuneus_R	3.32	0.003
DLPFC_L	Caudal temporal thalamus_L***^a^***	3.19	0.004
DLPFC_L	Posterior parietal thalamus_R***^a^***	3.74	<0.001
DLPFC_L	Lateral prefrontal thalamus_R***^a^***	3.08	0.005
DLPFC_L	Cerebelum_6_L	4.38	<0.001
Temporal_Pole_Sup_R	Cerebelum_Crus1_R	3.44	0.002
**DSZ group (*t2* vs. *t1*)**			
DLPFC_L	Occipital_Sup_R	3.25	0.003
DLPFC_L	Cerebelum_6_L	3.13	0.004
DLPFC_L	Cerebelum_Crus2_R	3.39	0.002
Rostral temporal thalamus_R	Rolandic_Oper_L	3.20	0.004

*TSZ, the group received rTMS together with antipsychotic drugs; DSZ, the group only received antipsychotic drugs; L, left; R, right. ^a^Region name according to human Brainnetome Atlas (Fan et al., 2016).*

### Relationship Between Functional Connectivity Temporal Variability Alterations and Clinical Features

**FIGURE 4:**
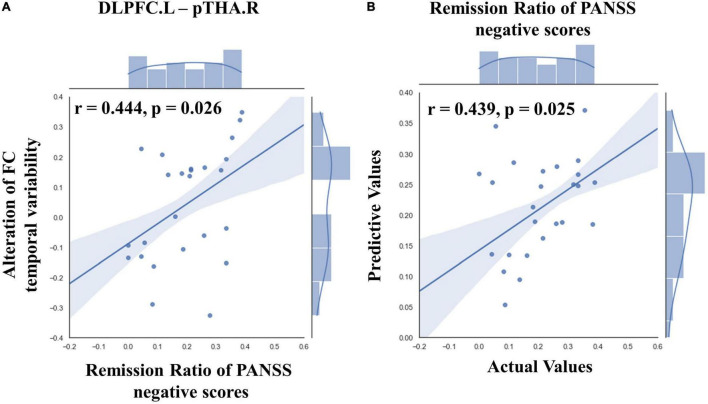
Results of ROI-wise functional connectivity (FC) temporal variability correlation analysis and prediction results of SVR in TSZ group. **(A)** The correlation analysis showed that the alteration in FC temporal variability between left DLPFC and right posterior parietal thalamus was significantly positively correlated with the remission ratio of Positive and Negative Syndrome Scale (PANSS) negative symptom scores. **(B)** The SVR result revealed a significantly positive relationship between the connection patterns at pre-rTMS intervention and the remission ratio of PANSS negative scores. The best parameters were obtained by grid search method (best *C* = 1,000, *g* = 0.01, MSE = 0.021). Values greater than 2 standard deviations were excluded as outliers. PANSS, Positive and Negative Syndrome Scale; DLPFC.L, left dorsolateral prefrontal cortex; pTHA.R, right posterior parietal thalamus.

Our results showed that after high-frequency rTMS intervention, the alteration in FC temporal variability between left DLPFC and right posterior parietal thalamus was significantly positively correlated with the remission ratio of PANSS negative symptom scores (*r* = 0.444, *p* = 0.026, Figure 4A).

### Pattern Classification Results

In the TSZ group, the paired *t*-test was used to identify ROI-wise FC temporal variability differences between time points *t1* and *t2*. Using a relatively high threshold of *p* < 0.005, nine FC temporal variability patterns with significant differences were retained (Figure 3). We hypothesized that the connection patterns of the FC temporal variability were related to the treatment response to high-frequency rTMS. To test the possibility, an SVR prediction model was constructed based on the connection pattern. The SVR result revealed a significantly positive relationship between the connection pattern at *t1* and the remission rate of PANSS negative scores (*r* = 0.439, *p* = 0.025, Figure 4B). No significant relationship was found between the connection pattern at *t1* and the remission rates of PANSS positive, general, and total scores (Supplementary Figure 4).

## Discussion

This study adopts a longitudinal experimental design to comprehensively evaluate the changing patterns of the functional dynamics of patients with schizophrenia before and after the treatment with high-frequency rTMS at left DLPFC, and illuminated the alterations in FC temporal variability in the brain circuit of patients with schizophrenia induced by high-frequency rTMS and its relationship with clinical manifestations. First, the voxel-wise FC temporal variability of the high-frequency rTMS target showed significant interaction effects of treatment and time between the left DLPFC (target) and brain regions, including the thalamus, cerebellum, and several cortical regions, which are mainly located within the cortico-thalamo-cerebellar circuit. Further, the ROI-wise analysis demonstrated a significant interaction effect on the FC temporal variability within the cortex and CTCC. The *post-hoc* test showed that the FC temporal variability between the target and regions in CTCC was significantly reduced after high-frequency rTMS intervention, and decreased FC temporal variability was also found in the regions of CTCC. In addition, higher alteration in FC temporal variability between left DLPFC and right posterior parietal thalamus predicted a higher remission ratio of negative symptom scores, indicating that the decrease of FC temporal variability between brain regions was associated with the remission of schizophrenia severity. Meanwhile, SVR results suggest that the baseline pattern of FC temporal variability between brain regions can predict the efficacy of high-frequency rTMS intervention on negative symptoms in patients with schizophrenia. In conclusion, the time-varying approach may reveal the dynamic brain activity patterns induced by high-frequency rTMS. Our findings support that high-frequency rTMS regulates the FC temporal variability in CTCC in patients with schizophrenia, which was associated with symptomatology improvement.

As a physiotherapy strategy different from drug therapy, rTMS may have a broad application prospect in schizophrenia for the treatment of auditory hallucinations, negative symptoms, and cognitive deficits, but the effects were mixed (Kennedy et al., 2018; Brandt et al., 2021). The inability of the field to reach a consensus on the use of rTMS in schizophrenia stems from a variety of issues. Specifically, different stimulation patterns were observed to result in varying physiologic effects (Hamada et al., 2008; Jannati et al., 2019; Mehta et al., 2019; Tiksnadi et al., 2020) and the significant heterogeneity in the participants across different studies. In our study, most of the patients with schizophrenia predominantly had a negative symptom profile patients. After 4 weeks of high-frequency rTMS, the scores of each PANSS subscale in the TSZ group were significantly decreased. At the same time, the PANSS scale scores of the DSZ group also decreased significantly. Considering the time distance between the two PANSS assessments in the current experimental design, we speculate that a shorter or longer time distance design may help reveal the effects of TMS treatment. Of note, the remission ratios of PANSS negative symptoms, general psychopathology, and total scale scores in the TSZ group were more significant. Numerous studies had reported similar benefits of high-frequency rTMS on negative symptoms in patients with schizophrenia. A double-blind, sham-controlled, high-frequency rTMS study demonstrated that the rTMS group showed effective amelioration of negative symptoms, indicating that rTMS may have potential benefits in improving clinical symptoms and cognitive functions in patients with chronic schizophrenia (Wen et al., 2021). Moreover, a high-frequency (20 Hz) unilateral rTMS over the left DLPFC suggested that rTMS may lead to an improvement in the negative symptoms of schizophrenia (Kumar et al., 2020). In conclusion, high-frequency rTMS stimulation over the left DLPFC at a high stimulation intensity with a sufficient number of applied stimulating pulses may represent an efficient complement to antipsychotics in alleviating the negative symptoms of schizophrenia (Shi et al., 2014). This is consistent with our results that high-frequency rTMS combined with drug treatment can significantly improve the negative symptoms and general pathological symptoms of schizophrenia, indicating that high-frequency rTMS has a certain improvement effect on the psychiatric symptoms of patients with schizophrenia.

Using a time-varying approach, this study provides new insights into the changes in FC temporal variability induced by high-frequency rTMS in schizophrenia. Our results demonstrated that after high-frequency rTMS intervention, the voxel-wise FC temporal variability results found that the target showed no increase but a decrease in FC temporal variability in the regions of CTCC, including the thalamus, cerebellum, and several cortical regions in the TSZ group. These results may reflect the perturbation of high-frequency rTMS to FC temporal variability in CTCC. Similar to a previous study on FC temporal variability and strength coupling in schizophrenia (Deng et al., 2021), our study found a significant negative correlation between FC temporal variability and FC. Only decreased FC temporal variability was found in the TSZ group between left DLPFC and other brain regions after high-frequency rTMS intervention. This indicates that the decrease in FC temporal variability between the brain regions will lead to an increase in FC. In contrast, compared with baseline, the left DLPFC did not show a decrease but exhibited a significant increase in the FC temporal variability in VN and left precuneus in the DSZ group, which may imply a reduction in the FC. These results may suggest that drug therapy alone cannot counteract the disruption in the functional activity between the brains caused by schizophrenia. The DLPFC-related dysconnectivity may be related to the disturbance in the internal brain activity. Reduced neural integration between the DLPFC and the bilateral caudate, left middle/inferior frontal gyrus, left precentral gyrus, and right cerebellum had been found in patients with schizophrenia and their relatives. Deficits in brain coordination may contribute to impaired cognitive function in schizophrenia (Su et al., 2013). Combining previous work and the current novel findings, the high-frequency rTMS may harmonize the coactivation between DLPFC and the regions in CTCC by reducing the FC temporal variability, in an attempt to compensate for the FC reduction and activation imbalance of schizophrenia.

Furthermore, the results based on the ROI-wise FC temporal variability showed that the FC temporal variability between ROIs of patients with schizophrenia significantly decreased after high-frequency rTMS intervention in the TSZ group, while they increased in the DSZ group. The FC alterations between numerous brain regions, including frontal, temporal pole, parietal pole, subcortical and cerebellar, were thought to play an important role in the pathophysiology of schizophrenia (Jafri et al., 2008; Jones et al., 2012; Dong et al., 2019). On the other hand, abnormal FC temporal variability between different brain regions and networks in schizophrenia has been repeatedly reported in previous studies. A previous study based on independent component analysis confirmed a higher dynamic network configuration between a single independent component network and the other brain networks in schizophrenia. It has been reported that the activity in different brain networks may be weaker and more unstable in patients with schizophrenia, leading to higher variability between networks (Liu et al., 2006). Increased FC temporal variability and complexity in the heterogeneous functional organization are particularly critical for the understanding of schizophrenia, as it represents a highly relevant clinical representation of psychosis (Bassett et al., 2012). This greater brain network reconfiguration may be a manifestation of excessive neural variability. The high fluctuation of brain organization can lead to unstable communications between brain regions, thus affecting the perception of the environment. As a result, patients may have difficulty interacting with external stimuli, which may be clinically manifested as an increase in negative symptoms in patients with schizophrenia (Dinstein et al., 2015). Therefore, abnormal FC may ultimately manifest as an imbalance in the transmission of functional information between brain regions, which is accompanied by increased FC temporary variability. Thus, the reduction in FC temporal variability between brain regions following high-frequency rTMS in schizophrenia may be an underlying neuroregulatory mechanism for the reduction of negative symptoms.

Deficits in CTCC have been consistently observed in schizophrenia (Andreasen and Pierson, 2008; Picard et al., 2008). The loops that link the cerebellum with the cortical cortex have been considered to be anatomically connected through the thalamus. In general, thalamic dysconnectivity is a common abnormal mechanism in schizophrenia. This dysconnectivity was evident in people at high clinical risk for psychosis, and even more prominently in those who later converted to psychosis. These connectivity abnormalities have also been shown to correlate with symptom severity (Anticevic et al., 2015). Regulating abnormal connections between the thalamus and other brain regions may improve the severity of symptoms in schizophrenia. Of note, higher alteration in FC temporal variability between left DLPFC and right posterior parietal thalamus predicted a higher remission ratio of PANSS negative symptom scores, indicating that the decrease in FC temporal variability between brain regions was associated with the remission of schizophrenia severity. Similar to our previous work on the FC of thalamus subdivisions in schizophrenia, extensive functional dysconnectivity was found in the CTCC (Gong et al., 2019). Neil and his colleagues suggest that the changes in thalamic and prefrontal connectivity observed in schizophrenia may be due to abnormal late maturation of brain development during the transition from adolescence to adulthood, which disrupts the normal development of the prefrontal-thalamic connectivity (Woodward et al., 2012). The effectiveness of using the cerebellum as a stimulation target for rTMS in the treatment of schizophrenia has been demonstrated in several studies, and our results further demonstrate the pivotal role of the cerebellum (Demirtas-Tatlidede et al., 2010; Garg et al., 2016; van Dun et al., 2017). For instance, a recent study using the cerebellum as a target confirmed that cerebellum rTMS can modulate the severity of negative symptoms of schizophrenia by significantly improving the FC between the cerebellum and DLPFC, suggesting the impaired FC may be the underlying mechanism for negative symptoms in schizophrenia (Brady et al., 2019). In general, our findings verified the importance of quantifying FC temporal variability in CTCC, suggesting a possible neuro-mechanism for the beneficial effects of high-frequency rTMS.

Different from the traditional FC analysis, a novel dFC approach was adopted to explore the FC temporal variability between brain regions. Therefore, in order to emphasize the clinical value of this approach in high-frequency rTMS interventions, we used SVR to predict the efficacy of high-frequency rTMS interventions on symptoms in patients with schizophrenia. The SVR results indicated that the baseline pattern of FC temporal variability between these brain regions in CTCC with significant changes after intervention can predict the efficacy of high-frequency rTMS intervention on negative symptoms of schizophrenia. Hence, the established pre-treatment prediction can help psychiatrists make informed, individualized decisions about whether or not to intervene with rTMS when initiating the treatment.

There are certain limitations in this study. First, our study included a relatively small sample size. Second, the patients in the TSZ group received antipsychotic medications before and during the rTMS intervention, thereby introducing a confounding factor in the simple interpretation of our results. Third, although we had ensured that there were no significant differences with regard to gender, age, years of education, duration of illness, and dosage of antipsychotic drugs before grouping the patients, there were still significant differences in FC temporal variability between the TSZ and DSZ groups at baseline. Given that schizophrenia is a complex and heterogeneous syndrome, the FC temporal variability measurement in our study may be a potential biomarker for the subtype subdivision of schizophrenia. Additionally, our study lacked a sham TMS control group, which might have confused our results with placebo effects. Furthermore, we found that FC temporal variability in schizophrenia may predict the efficacy of high-frequency rTMS on negative symptoms, and we can further explore this conjecture using machine learning methods. Finally, our study indicates that high-frequency rTMS combined with antipsychotic therapy might contribute to the improvement of abnormal symptomatology via inducing a reconfiguration of FC temporal variability in patients with schizophrenia, perhaps in relation to the underlying neural mechanism that involves the activation of brain function by high-frequency rTMS.

## Conclusion

In summary, our study confirmed that high-frequency rTMS could induce reduced FC temporal variability in schizophrenia. The rTMS effect cannot only affect the FC temporal variability between the rTMS target and regions in CTCC, but also alter the FC temporal variability among regions in CTCC. Furthermore, the reduced FC temporary variability was correlated with the individual therapeutic response in patients with schizophrenia. These findings confirm the potential relationship between the reduction in whole-brain FC temporary variability induced by high-frequency rTMS and the improvement in schizophrenia symptoms, suggesting that high-frequency rTMS affects psychiatric symptoms by coordinating the heterogeneity of activity between brain regions. Future studies are warranted to further examine the clinical utility of FC temporal variability patterns between specific brain regions as a biomarker to predict treatment response to high-frequency rTMS in patients with schizophrenia.

## Data Availability Statement

The raw data supporting the conclusions of this article will be made available by the authors, without undue reservation.

## Ethics Statement

The studies involving human participants were reviewed and approved by Clinical Hospital of Chengdu Brain Science Institute. The patients/participants provided their written informed consent to participate in this study.

## Author Contributions

DY and CLu designed the study and supervised the project. XC, YL, LM, CLi, and MD managed the experiments and data collection. HHu, BZ, HHe, JZ, ML, HL, RY, and QL undertook the data analysis. HHu, BZ, SJ, and CLu wrote and revised the manuscript. All authors reviewed the manuscript and approved the final manuscript.

## Conflict of Interest

The authors declare that the research was conducted in the absence of any commercial or financial relationships that could be construed as a potential conflict of interest.

## Publisher’s Note

All claims expressed in this article are solely those of the authors and do not necessarily represent those of their affiliated organizations, or those of the publisher, the editors and the reviewers. Any product that may be evaluated in this article, or claim that may be made by its manufacturer, is not guaranteed or endorsed by the publisher.

## Footnotes

1
https://scikit-learn.org/
