# Supplementary material for: Reconfiguration of Functional Dynamics in Cortico-Thalamo-Cerebellar Circuit in Schizophrenia Following High-Frequency Repeated Transcranial Magnetic Stimulation
Source: Front Hum Neurosci. 2022 Jul 25;16:928315. doi: 10.3389/fnhum.2022.928315 (PMC9359206; doi:10.3389/fnhum.2022.928315)
Supplement: Supplementary file 1 [file Data_Sheet_1.docx]

**Supplementary Material For**

**Reconfiguration of Functional dynamics in**

**Cortico-thalamo-cerebellar Circuit in schizophrenia following high-frequency repeated transcranial magnetic stimulation**

***This supplementary material includes as follows:***

**PART 1: Methods and Materials**

**Methods and Materials:** ***Supplementary Analysis***

**PART 2: Supplementary Figures and Tables**

**Fig S1:** ***Negative associations between FC Temporal Variability and FC across all the ROIs at group-level.***

**Fig S2:** ***Regions with significant differences voxel-wise FC Temporal Variability between SZ patients in the TSZ group and HC.***

**Fig S3:** ***Regions with significant differences voxel-wise FC Temporal Variability between SZ patients in the DSZ group and HC.***

**Fig S4:** ***Correlation results between the predicted value and the real value for remission ratios of PANSS scores.***

**Tables S1: *ROIs abbreviation corresponding table***

**Methods and Materials**

***Supplementary Analysis***

For the ROI-wise FC, the time course of each ROI was defined as the mean of the time courses of peak value voxel and its nearest 6 neighbors. Further, the FC was calculated as the Pearson’s correlation coefficient between the BOLD time course of each pair of ROIs. Then, for each subject, a *m*x*m* (*m* = number of ROIs) FC matrix was generated. To further illustrate whether the FC temporal variability change was accompanied with increased or decreased FC, the correlation between FC temporal variability and FC was analyzed. Specifically, for each subject, the coupling between FC temporal variability and FC was calculated as the correlation coefficient between FC temporal variability and FC across all the ROIs.

**Supplementary Figures and Tables**

**
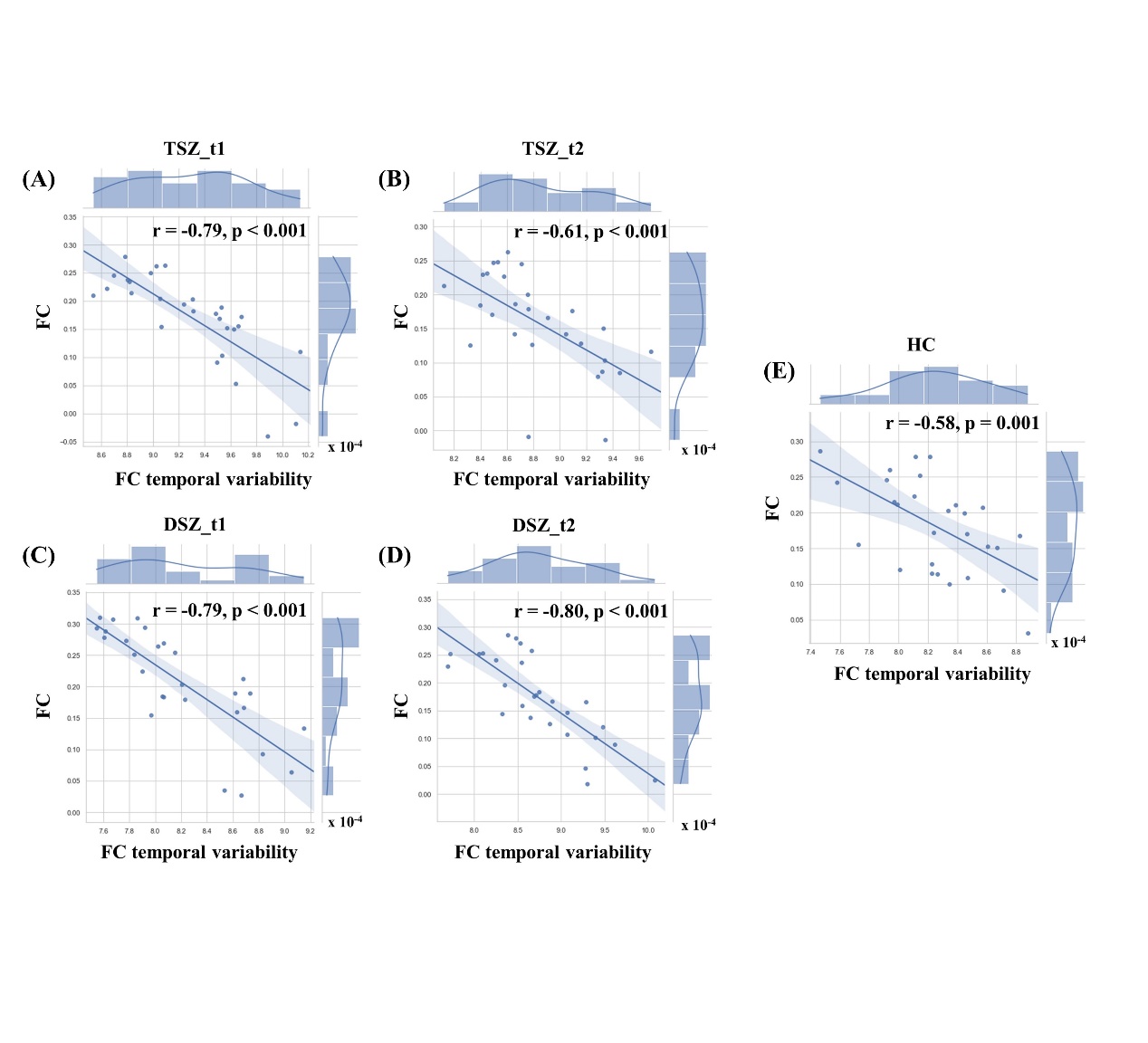
**

**Fig.S1** **Negative associations between FC temporal variability and FC across all the ROIs at group-level.** (A) The TSZ group at t1. (B) The TSZ group at t2. (C) The DSZ group at t1. (D) The DSZ group at t2. (E) The HC group at t1. To show the group-level associations between FC temporal variability and FC, each dot in (A)-(E) represents the average values of the corresponding group for a single ROI, from all ROIs. ROIs, regions of interest. FC, functional connectivity. TSZ group, the group received rTMS together with antipsychotic drugs. DSZ group, the group only received antipsychotic drugs. t1 and t2, the baseline and after 4-week treatment.

**
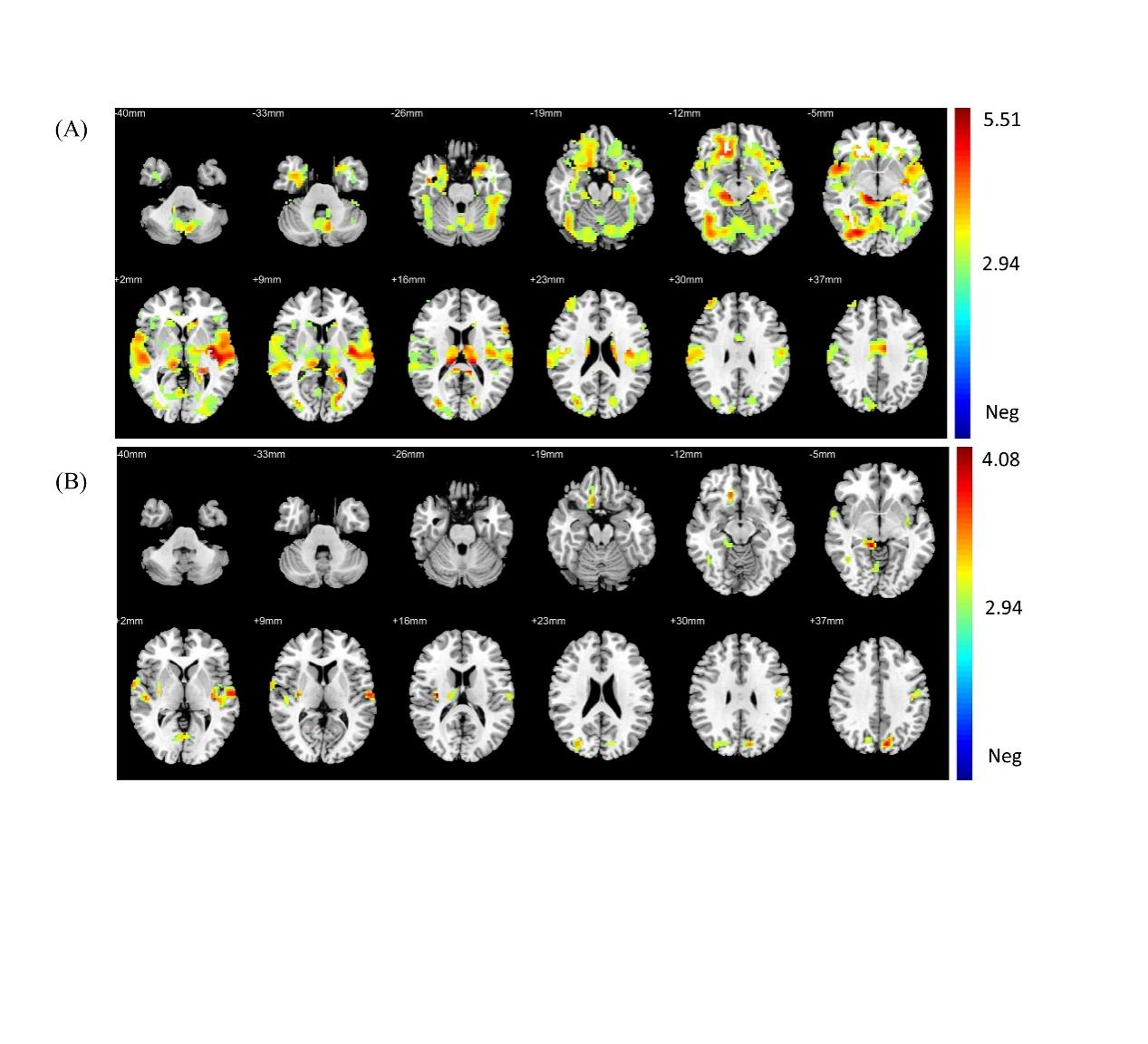
**

**Fig.S2 Regions with significant differences voxel-wise FC temporal variability between SZ patients in the TSZ group and HC.** (A) and (B) are the results of t1 and t2 respectively. Two-sample t-test was performed, p < 0.005, clusters size > 26. FC, functional connectivity. SZ, schizophrenia. TSZ, the group received rTMS together with antipsychotic drugs. HC, healthy controls. t1 and t2, the baseline and after 4-week treatment.


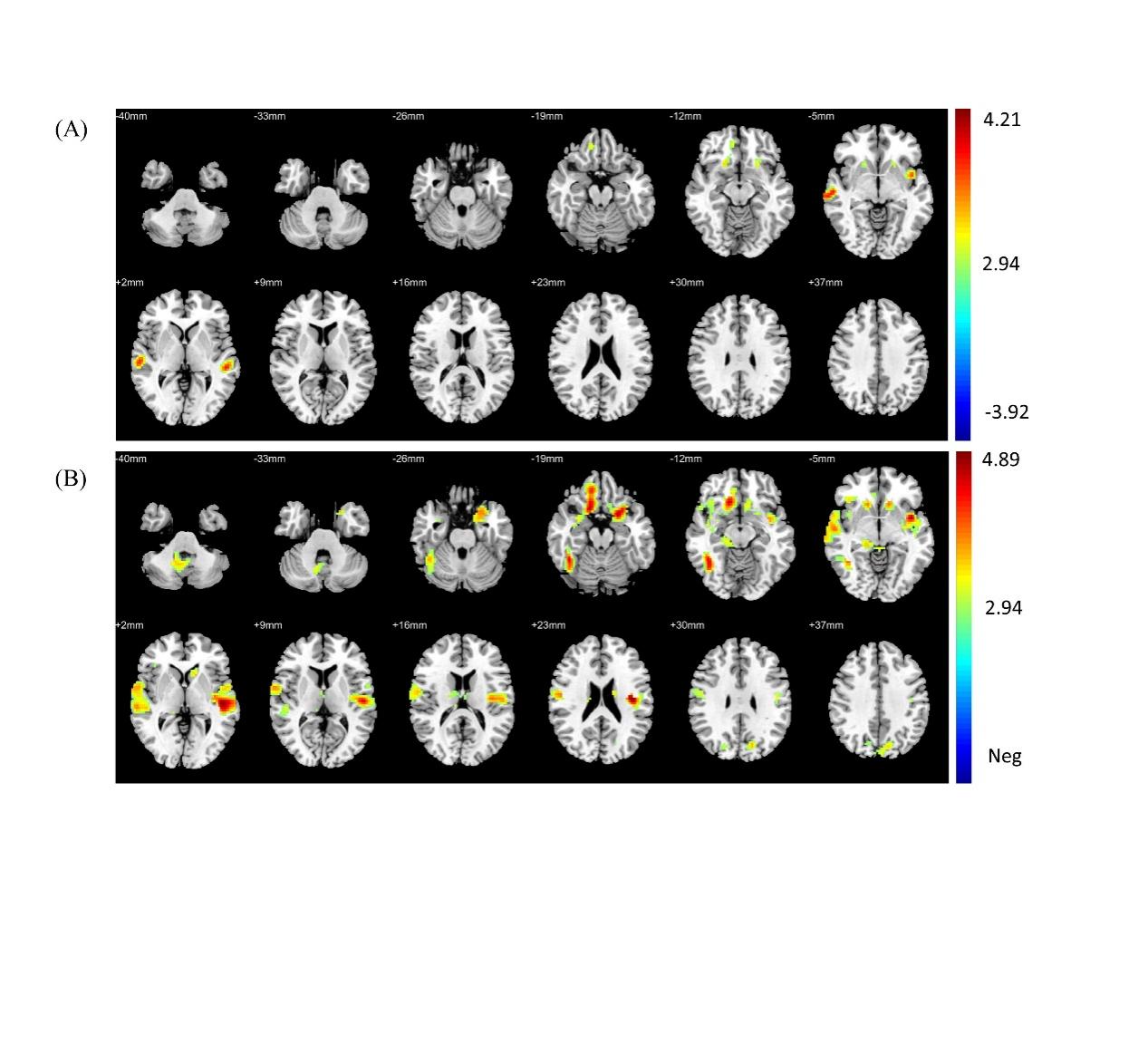


**Fig.S3 Regions with significant differences voxel-wise FC temporal variability between SZ patients in the DSZ group and HC**. (A) and (B) are the results of t1 and t2 respectively. Two-sample t-test, p < 0.005, clusters size > 26. FC, functional connectivity. SZ, schizophrenia. DSZ, the group only received antipsychotic drugs. HC, healthy controls. t1 and t2, the baseline and after 4-week treatment.

**
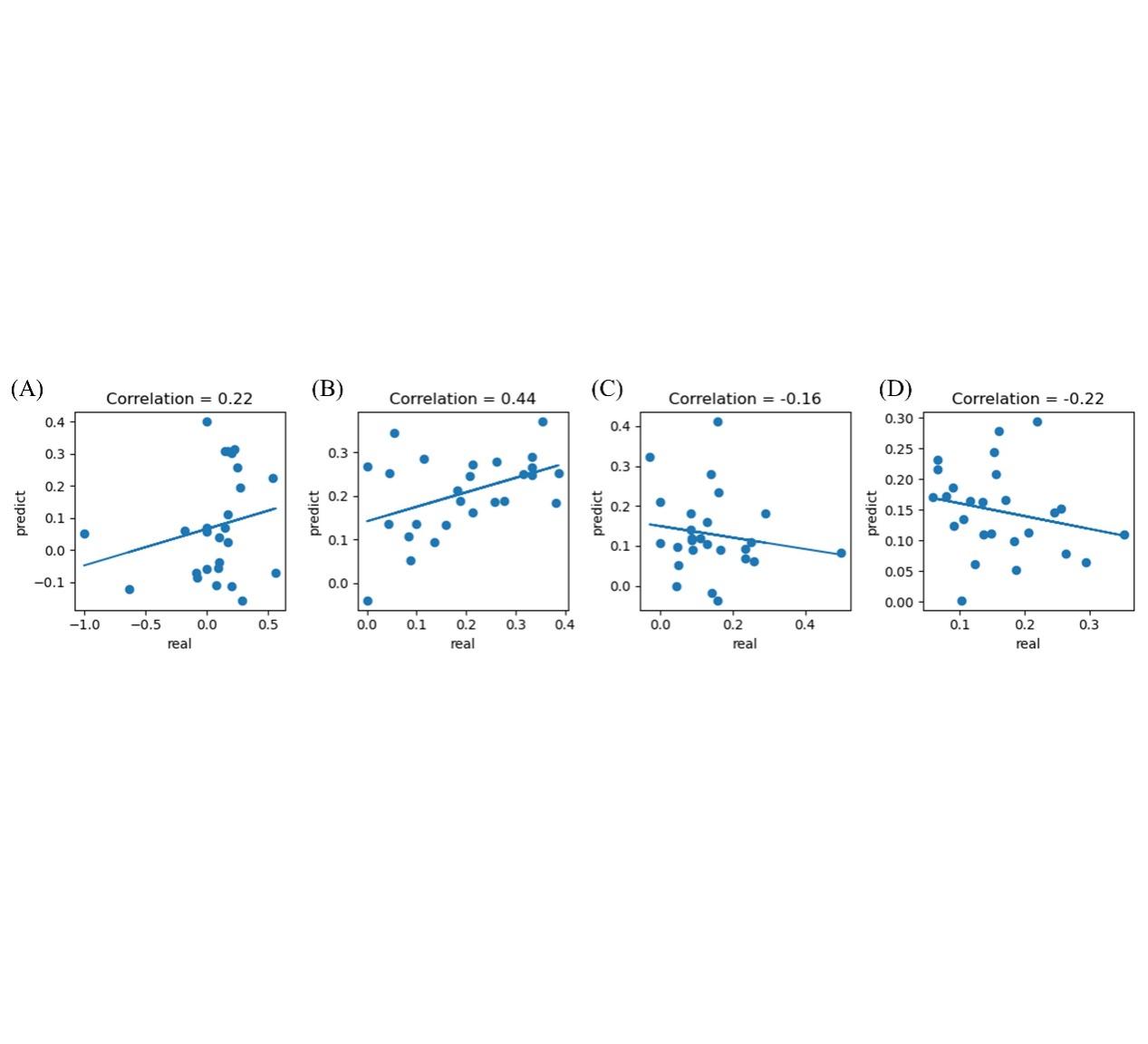
**

**Fig.S4** **Correlation results between the predicted value and the real value for remission ratios of PANSS scores**. (A)-(D) represents the remission ratio of PANSS of positive, negative, general and total scores, respectively. The SVR result revealed a significantly positive relationship between the connection pattern at t1 and the remission ratio of PANSS negative scores (r = 0.44, p = 0.026). No significant relationship was found between the connection pattern at t1 and the remission ratios of PANSS positive, general and total scores. t1, the baseline before the rTMS.

**Tables S1: *ROIs abbreviation corresponding table***

| **Brain Region** | **abbreviation** |
| --- | --- |
| **Cortical** **(AAL)** |  |
| Temporal_Inf_L | ITG.L |
| Fusiform_L | FG.L |
| Precuneus_L | PCUN.L |
| Cingulum_Ant_L | ACC.L |
| ParaHippocampal_L | PHIP.L |
| Rolandic_Oper_L | ROL.L |
| Frontal_Inf_Orb_R | IFGorb.R |
| Precuneus_R | PCUN.R |
| ParaHippocampal_R | PHIP.R |
| Hippocampal_R | HIP.R |
| Postcentral_R | POCG.R |
| Paracentral_Lobule_R | PCL.R |
| Occipital_Sup_R | SOG.R |
| Temporal_Pole_Sup_R | STGp.R |
| Temporal_Pole_Mid_R | MTGp.R |
| **Thalamus****^a^** |  |
| rostral temporal thalamus_L | rTHA.L |
| caudal temporal thalamus_L | cTHA.L |
| rostral temporal thalamus_R | rTHA.R |
| caudal temporal thalamus_R | cTHA.R |
| posterior parietal thalamus_R | pTHA.R |
| lateral prefrontal thalamus_R | lTHA.R |
| **Cerebellum (AAL)** |  |
| Vermis_3 | Vermis.3 |
| Vermis_4_5 | Vermis.4.5 |
| Cerebelum_6_L | Cere6.L |
| Cerebelum_9_L | Cere9.L |
| Cerebelum_Crus1_R | CCrus1.R |
| Cerebelum_Crus2_R | CCrus2.R |

Note: AAL, automated anatomical labeling atlas. **^a^**Human Brainnetome Atlas [1].

**References**

[1] Fan, L., Li, H., Zhuo, J., Zhang, Y., Wang, J., Chen, L., Yang, Z., Chu, C., Xie, S., Laird, A. R., Fox, P. T., Eickhoff, S. B., Yu, C. and Jiang, T. The Human Brainnetome Atlas: A New Brain Atlas Based on Connectional Architecture. *Cereb Cortex*, 26, 8 (Aug 2016), 3508-3526.
